# Classification of selected cardiopulmonary variables of elite athletes of different age, gender, and disciplines during incremental exercise testing

**DOI:** 10.1186/s40064-015-1341-8

**Published:** 2015-09-24

**Authors:** Christoph Zinner, Billy Sperlich, Patrick Wahl, Joachim Mester

**Affiliations:** Institute of Training Science and Sport Informatics, German Sport University Cologne, Am Sportpark Müngersdorf 6, 50933 Cologne, Germany; The German Research Centre of Elite Sport, Am Sportpark Müngersdorf 6, 50933 Cologne, Germany; Department of Sport Science, Julius-Maximilians-University Würzburg, Judenbühlweg 11, 97082 Würzburg, Germany

**Keywords:** Competition, Data, Performance, Reference values

## Abstract

Incremental exercise testing is frequently used as a tool for evaluating determinants of endurance performance. The available reference values for the peak oxygen uptake (VO_2peak_), % of VO_2peak_, running speed at the lactate threshold (v_LT_), running economy (RE), and maximal running speed (v_peak_) for different age, gender, and disciplines are not sufficient for the elite athletic population. The key variables of 491 young athletes (age range 12–21 years; 250 males, 241 females) assessed during a running step test protocol (2.4 m s^−1^; increase 0.4 m s^−1^ 5 min^−1^) were analysed in five subgroups, which were related to combat-, team-, endurance-, sprint- and power-, and racquet-related disciplines. Compared with female athletes, male athletes achieved a higher v_peak_ (P = 0.004). The body mass, lean body mass, height, abs. VO_2peak_ (ml min^−1^), rel. VO_2peak_ (ml kg^−1^ min^−1^), rel. VO_2peak_ (ml min^−1^ kg^−0.75^), and RE were higher in the male participants compared with the females (P < 0.01). The % of VO_2_ at v_LT_ was lower in the males compared with the females (P < 0.01). No differences between gender were detected for the v_LT_ (P = 0.17) and % of VO_2_ at v_LT_ (P = 0.42). This study is one of the first to provide a broad spectrum of data to classify nearly 500 elite athletes aged 12–21 years of both gender and different disciplines.

## Background

Cardiopulmonary exercise testing is frequently used as a tool for evaluating endurance performance and cardiopulmonary fitness in elite athletes. During graded exercise testing performed to voluntary maximal exertion, several sub-maximal and maximal physiological variables are assessed, which enables to: (1) assess the cardiopulmonary capacity; (2) explain the possible mechanisms of reduced exercise tolerance; and (3) to compare inter- or intra-individual differences among various disciplines, ages, and different genders.

Five major variables have been described to explain the inter-individual variance in aerobic endurance performance (Midgley et al. 2007). The VO_2peak_ represents one of the most important parameters for endurance performance and has been extensively assessed among individuals of different age, gender, and performance levels. VO_2peak_ is directly influenced by maximal cardiac output and arterio-venous oxygen difference, and therefore has become a standard in cardiopulmonary exercise testing (Bassett and Howley 2000).

During graded exercise testing, some variables demonstrate intensity-dependent benchmarks, such as the level of blood lactate, which have led to the development of numerous lactate threshold (LT) concepts (Faude et al. 2009). In endurance athletes, previous studies have demonstrated that the velocity at the lactate threshold (v_LT_) is closely linked to endurance performance (Bassett and Howley 2000; Midgley et al. 2007). As a result, the v_LT_ and fractional utilisation of VO_2_ at v_LT_ (% VO_2peak_) are regarded as good indicators of endurance performance between individuals of different age, gender, and disciplines (Midgley et al. 2007; Ziogas et al. 2011). Running economy [RE; defined as the steady state VO_2_ expressed in ml kg^−1^ min^−1^ at a standard velocity (Costill et al. 1973), or as the energy cost of running per meter (ml min^−1^ m^−1^) (di Prampero et al. 1986)] differentiate among athletes of different performance levels (Conley and Krahenbuhl 1980). Finally, peak treadmill running velocity (v_peak_) has been previously described as a good predictor of endurance performance (Noakes et al. 1990).

Thus, it can be assumed that the VO_2peak_, % of VO_2peak_, v_LT_, RE, and v_peak_ are important in the comparison of endurance performance amongst well-trained individuals, and the characteristics of these variables are of particular interest for elite athletes of different age, gender, and performance levels. From a practical point of view, information about the aforementioned determinants of endurance performance may aid in distinguishing individual differences in cardiopulmonary fitness, as well as designing proper conditioning programs for athletes of different age, gender, and disciplines. Therefore, the aims of this study in elite athletes of different disciplines, age, and gender were: (1) to investigate the absolute and relative VO_2peak_, % of VO_2peak_, v_LT_, RE, and v_peak_; and (2) to classify selected cardiopulmonary variables.

## Methods

### Participants and design

A total of 550 athletes participating in the health and physical fitness monitoring program of the German Research Centre of Elite Sport between September 2006 and December 2011 were examined with an incremental test to exhaustion. All participants and/or their guardians gave their written informed consent to participate in this study. All procedures were approved by the Ethical Committee of the German Sport University Cologne and conducted in accordance with the Declaration of Helsinki. All participants were fully familiarised with the laboratory exercise procedures prior to data collection. None of the participants demonstrated any pathological pulmonary or cardiac findings during the pre-medical examination.

The inclusion criterion for participation in this program was to have an active status within the elite A, B, and C squad system of the German Olympic Federation. In Germany, this squad system is used to define the performance level of an athlete. Depending on the discipline, A-level athletes have achieved top-level positions at Olympic Games or World Championships, B-level athletes displayed considerable performance development and are prospective candidates for A-level status, and C-level included the highest national level for prospective young athletes who exhibit the potential to perform at the top international level or who competed successfully in international junior competitions.

A total of 491 athletes (250 males and 241 females) were included into the analyses since the others did not meet the criteria for maximal exhaustion. According to the general physiological characteristics of their disciplines, all athletes were grouped into five subgroups, which were related either to combat, team, endurance, sprint and power, or racquet sports, as described by previous researchers (Koehler et al. 2012). Please refer to Table 1 for the number of participants in each group, discipline, gender, age, and anthropometric data.

**Table 1:** The number of participants of each group and gender as well as their age and anthropometric data

Group	N		Age (years)				Height (cm)				Body mass (kg)			
	♀	♂	♀		♂		♀		♂		♀		♂	
			Mean	95 % CI	Mean	95 % CI	Mean	95 % CI	Mean	95 % CI	Mean	95 % CI	Mean	95 % CI
“Combat”	29	45	17.3 ± 4.3	15.7–18.9	16.9 ± 3.5	15.8–17.9	163.7 ± 7.6	160.8–166.6	177.4 ± 14.3	173.1–181.7	53.9 ± 10.2	50.0–57.7	70.5 ± 17.0	65.4–75.6
“Team”	62	42	16.1 ± 2.4	15.4–16.6	15.9 ± 1.7	15.4–16.5	170.7 ± 9.3	168.3–173.0	183.5 ± 7.6	181.1–185.8	60.9 ± 9.2	58.5–63.1	77.0 ± 13.1	73.0–81.0
“Endurance”	39	74	16.2 ± 2.3	15.4–16.9	16.5 ± 3.4	15.6–17.2	167.5 ± 6.2	165.5–169.5	177.8 ± 11.2	175.1–180.4	57.0 ± 9.1	54.0–59.9	67.7 ± 14.2	64.3–71.0
“Sprint & power”	73	53	17.0 ± 3.7	16.1–17.9	18.4 ± 3.9	17.3–19.4	165.4 ± 12.0	162.5–168.1	181.4 ± 9.0	178.9–183.8	55.5 ± 13.1	52.4–58.5	75.7 ± 13.2	71.8–79.1
“Racquet”	38	36	17.6 ± 4.3	16.2–19.0	16.8 ± 3.3	15.7–17.9	170.4 ± 5.2	168.6–172.0	180.3 ± 8.4	177.4–183.0	60.1 ± 7.2	57.7–62.4	68.7 ± 8.8	65.6–71.6
Total	241	250	16.8 ± 3.4	16.3–17.2	16.9 ± 3.4	16.5–17.3	167.7 ± 9.5	166.4–168.8	179.8 ± 10.7	178.4–181.1	57.7 ± 10.6	56.3–59.0	71.6 ± 14.2	69.8–73.3

Data are expressed as mean ± SD and 95 % CI

The “combat” group consisted of the disciplines of boxing (n = 26), judo (n = 25), fencing (n = 11), taekwondo (n = 8) and wrestling (n = 4). The “team” group consisted of soccer (n = 39), handball (n = 30), water polo (n = 16), basketball (n = 14), volleyball (n = 3) and field hockey (n = 2). The “endurance” group included canoeists (n = 29); kayakers (n = 17); 800 m (n = 1), 1500 m (n = 1), 10,000 m (n = 2) and 3000 m steeple (n = 2) runners; rowers (n = 24); biathletes (n = 13); triathletes (n = 12); nordic combined athletes (n = 8); modern pentathletes (n = 2) and swimmers (n = 2). The “sprint and power” group included 100 m (n = 5), 200 m (n = 5) and 400 m (n = 3) sprinters; 100 m (n = 2) and 400 m (n = 3) hurdlers; decathletes/heptathletes (n = 5); hammer (n = 1), discus (n = 3) and javelin (n = 1) throwers; long (n = 5), triple (n = 2) and high (n = 2) jumpers; pole vaulters (n = 8); equestrians (n = 11); vaulters (n = 1); divers (n = 2); bobsledders (n = 3); lugers (n = 18); skeleton (n = 15); archers (n = 5); shooters (n = 1) and gymnasts (n = 25). The “racquet” group included tennis (n = 33), badminton (n = 36) and table tennis (n = 5) players

### Procedures

Prior to performance testing, the subject’s body mass and lean body mass were measured using a four-electrode bio impedance body scale (BC 418 MA, Tanita Corp., Tokyo, Japan). Thereafter, participants performed a step protocol to physical exhaustion beginning at 2.4 m s^−1^ and increasing 0.4 m s^−1^ every 5 min on a motorised running treadmill (Woodway PPS 55, Lörrach, Germany).

During the testing, all respiratory data were collected with an open breath-by-breath spirograph (ZAN600, nSpire Health, Oberthulba, Germany) using standard algorithms to dynamically account for the time delay between the gas consumption and the volume signal. The system was calibrated before each test with a calibration gas (15.8 % O_2_, 5 % CO_2_ in N; Praxair, Germany) and a precision 3-l syringe (nSpire Health, Oberthulba, Germany). The respiratory variables were averaged every 30 s. The highest 30-s value of VO_2_ during the test was considered to be VO_2peak_.

The heart rate of each participant was recorded every 5 s during testing using short-range telemetry (Polar S 810, Kempele, Finland). All respiratory and heart rate data were averaged every 30 s and used for further analyses. A 20-µl blood sample from the right ear lobe was collected immediately after each step as well as immediately following the termination of the test. All samples were analysed amperometric-enzymatically (Ebio Plus, Eppendorf AG, Hamburg, Germany).

The RE was calculated using the average oxygen uptake of the last 30 s at 3.2 m s^−1^. This speed was chosen as it was below 85 % of the VO_2peak_ for all subjects, which is required to assess RE (Saunders et al. 2004). The v_LT_ was identified as the first significant elevation of blood lactate above baseline levels (at 2 mmol l^−1^), as described previously (Skinner and McLellan 1980).

For classification of each variable, five levels were selected according to Heyward (2010): superior > mean value + 2 standard deviation (SD); excellent > mean + SD; good > mean + 0.5 SD; fair > mean − 0.5 SD; poor < mean − SD.

### Statistical analysis

All data were calculated with conventional procedures and are presented as mean values and standard deviations or as odds ratios with 95 % confidence intervals (CI). All data were checked for normality, with no data necessary for further transformation. For the comparison of the different variables between age, sport, and gender, a multiple-measures ANOVA with Tukey post hoc test was used. An alpha of *p* < 0.05 was used for statistical significance. Where required, the effect size Cohen’s *d* was calculated to compare the variables. The thresholds for small, moderate, and large effects were defined as 0.20, 0.50 and 0.80, respectively (Cohen 1988). All calculations were processed using the Statistica (version 7.1, StatSoft Inc., Tulsa, OK, USA) software package for Windows^®^.

## Results

**Fig. 1:**
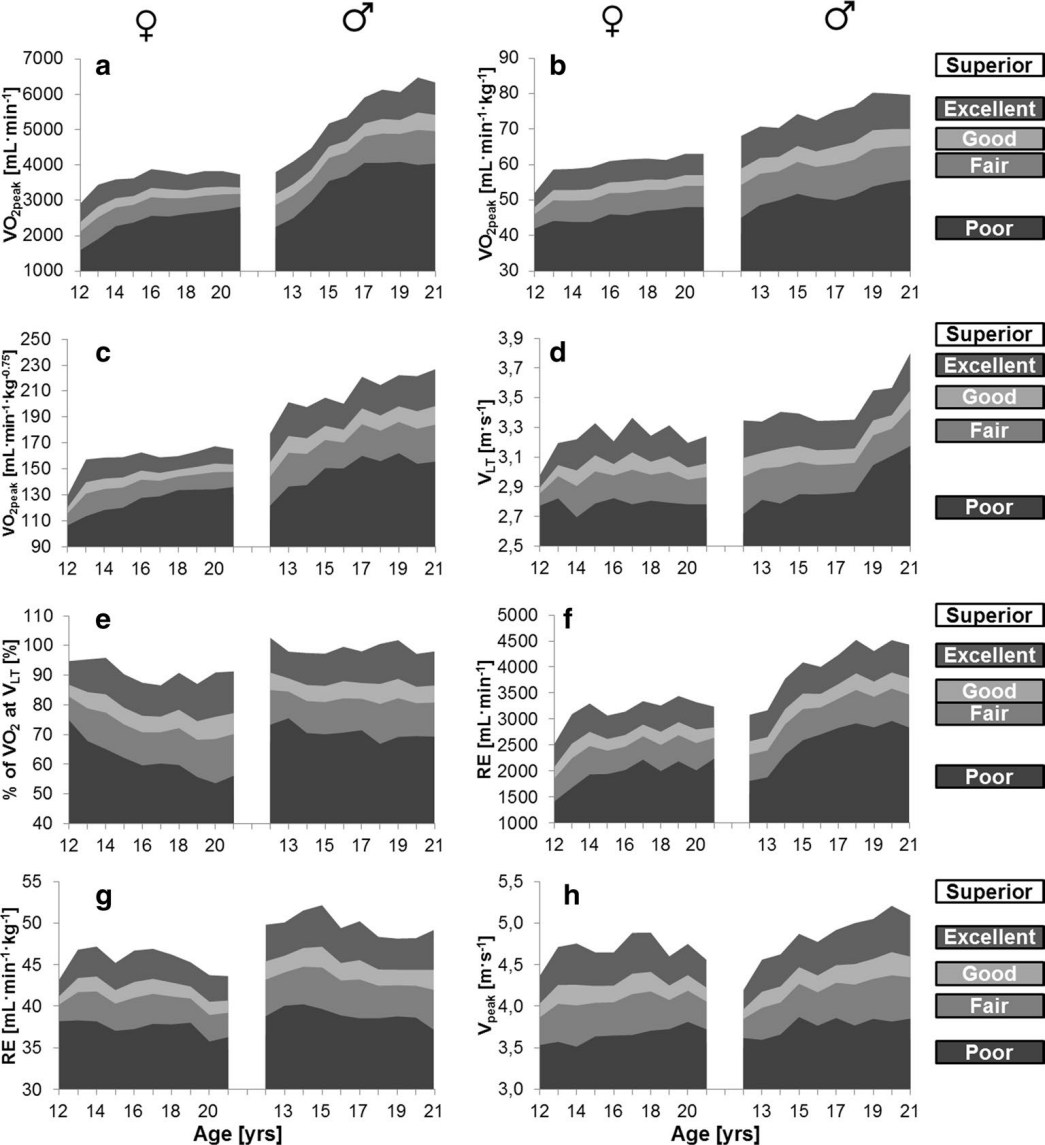
Reference values (superior, excellent, good, fair, poor) for both gender and age for **a** absolute oxygen uptake (ml min^−1^), **b** relative oxygen uptake (ml min^−1^ kg^−1^), **c** relative oxygen uptake (ml min^−1^ kg^−0.75^), **d** v_LT_ (m s^−1^), **e** % of VO_2_ at v_LT_ (%), **f** RE (ml min^−1^), **g** RE (ml min^−1^ kg^−1^), **h** v_peak_ (m s^−1^)

The exercise duration for all athletes averaged 22:36 ± 0:25 min. The male athletes achieved a higher v_peak_ (*p* = 0.004) in comparison with the females. The body mass, lean body mass, height, absolute VO_2peak_ (ml min^−1^), relative VO_2peak_ (ml kg^−1^ min^−1^), relative VO_2peak_ (ml min^−1^ kg^0.75^), and RE were higher in the male participants compared with the female participants (*p* < 0.01). The % of VO_2peak_ at v_LT_ was lower in the males compared with the females (*p* < 0.01). No differences between the gender were detected for v_LT_ (*p* = 0.17) (Fig. 1).

Peak values for blood lactate, heart rate, and respiratory exchange ratio did not differ between gender. All data from the maximal incremental test are presented in Table 2.

**Table 2:** Peak values for maximum running speed, blood lactate, heart rate, and respiratory exchange ratio (♀ n = 241, ♂ n = 250)

Peak variable	♀		♂		*P*
	Mean ± SD	95 % CI	Mean ± SD	95 % CI	
v_peak_ (m s^−1^)	3.86 ± 0.41	3.81–3.92	3.98 ± 0.45	3.92–4.04	0.004*
Peak blood lactate (mmol l^−1^)	5.87 ± 1.93	5.62–6.12	5.82 ± 2.02	5.57–6.07	0.79
Peak heart rate (beats min^−1^)	194 ± 8	193–195	193 ± 9	192–194	0.45
Peak respiratory exchange ratio	1.09 ± 0.07	1.08–1.10	1.08 ± 0.07	1.08–1.09	0.68

Data are expressed as mean ± SD and 95 % CI

**Fig. 2:**
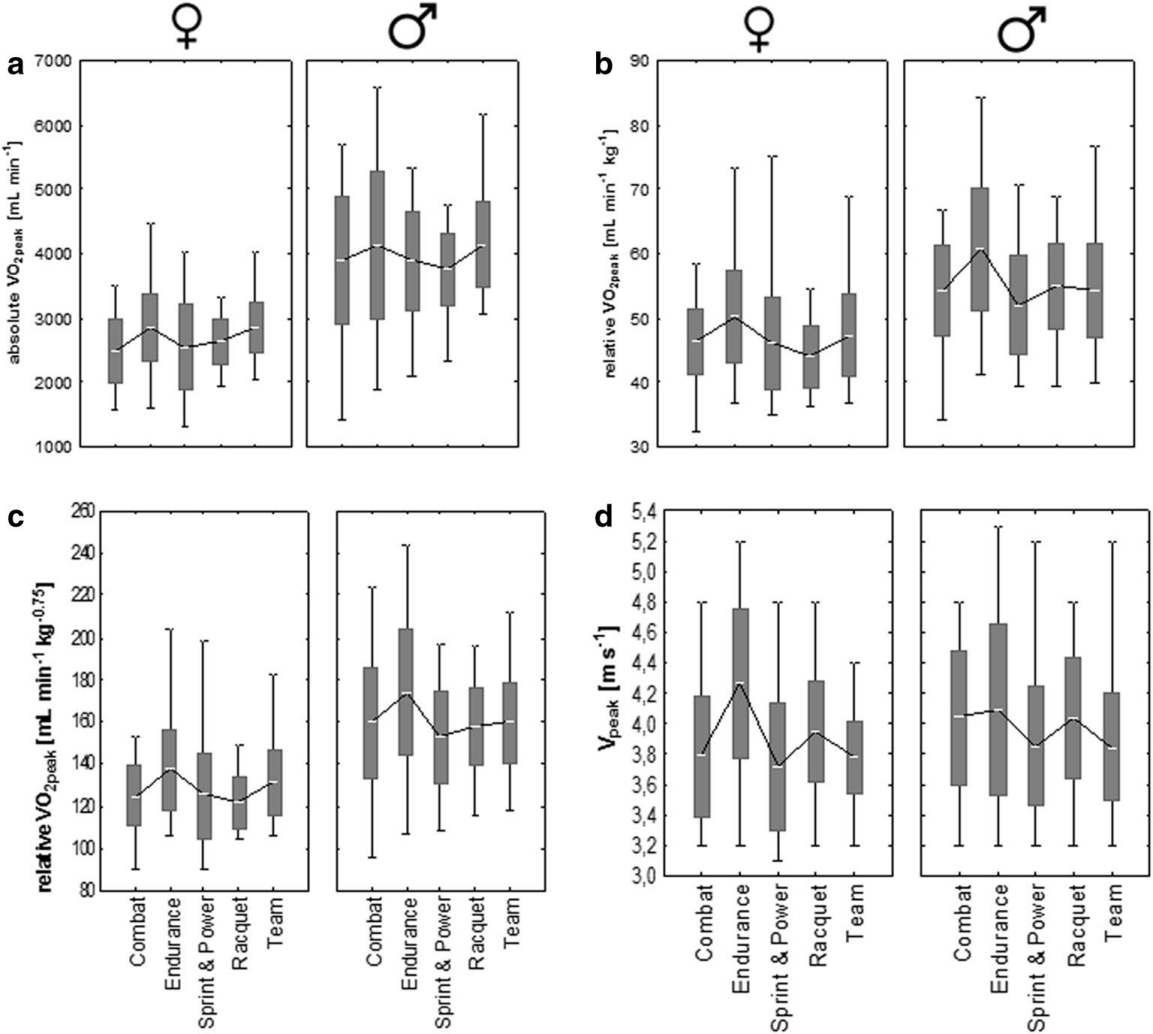
Overview of all mean ± SD and range data for all variables classified by discipline for both gender for **a** absolute oxygen uptake (ml min^−1^), **b** relative oxygen uptake (ml min^−1^ kg^−1^), **c** relative oxygen uptake (ml min^−1^ kg^−0.75^), **d** v_peak_ (m s^−1^). The *bars* are indicating standard deviation, the *whiskers* are indicating min and max values. *Asterisk* indicating significant differences between disciplines

**Fig. 3:**
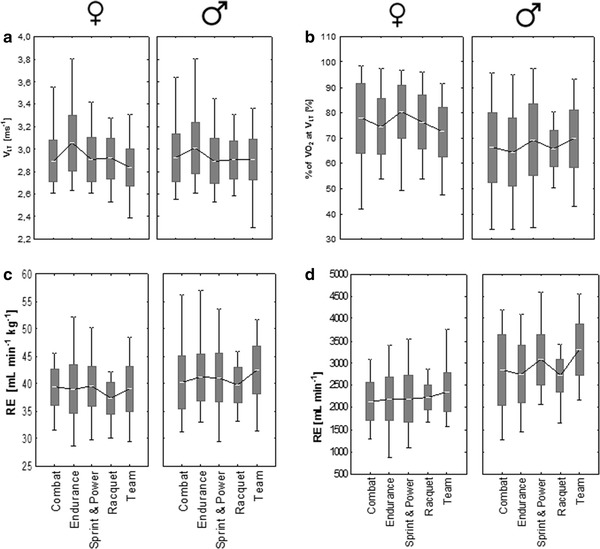
Overview of all mean ± SD and range data for all variables classified by discipline for both gender for **a**) v_LT_ (m s^−1^), **b** % of VO_2_ at v_LT_ (%), **c** RE (ml min^−1^ kg^−1^), **d** RE (ml min^−1^). The *bars* are indicating standard deviation, the *whiskers* are indicating min and max values. *Asterisk* indicating significant differences between disciplines

A summarised overview of all mean ± SD and range data for all variables and disciplines are presented in Figs. 2 and 3. Significantly higher values of % VO_2_ at v_LT_ (%) were found in the female combat, endurance, sprint & power, and racquet group when compared to males.

The body mass and lean body mass were lowest in the endurance group and highest in the team group for the males (*p* < 0.01; best d = 0.68). The relative VO_2peak_ (ml min^−1^ kg^−1^), relative VO_2peak_ (ml min^−1^ kg^−0.75^), v_LT_, and v_peak_ were the highest in the endurance group for both gender (*p* < 0.01; best d = 2.1). The RE (ml min^−1^) measured at a speed of 3.2 m s^−1^ was highest for both gender in the team-related disciplines compared with the other groups, with no differences for the RE (ml kg^−1^ min^−1^). The % of VO_2_ at v_LT_ was lowest in the female team group and the male endurance group (*p* < 0.01).

## Discussion

The primary aim of this study was to classify selected cardiopulmonary variables in elite athletes of different disciplines, age, and gender. The mean absolute VO_2peak_ (ml min^−1^) in this study increased steadily from age 12 to 21 years in both gender, with overall higher values in the male athletes. Similar results were observed in 175 healthy school children (8–18 years), with increased VO_2peak_ over age in boys, though this was not found in girls (Ten Harkel et al. 2011). A lower VO_2peak_ relative to body mass (ml min^−1^ kg^−1^) in girls in the 8- to 16-year age group has usually been attributed to an increase in body fat mass (Mota et al. 2002) and a lower relative amount of lean body mass. Therefore, the higher amount of fat-free mass during standard running treadmill testing would account for the higher need for oxygen utilisation and would explain the higher VO_2peak_ in boys compared with girls. Differences in VO_2peak_ between the gender have been reported to range from 8 to 63 %, with higher values for male athletes (Sandbakk et al. 2012; Seiler et al. 2007). In the current cross-sectional investigation, we measured 33 % higher mean absolute VO_2peak_ (ml min^−1^) values in male athletes compared with females. Previous investigations have attributed the main gender-related differences in the VO_2peak_ to lower body fat and higher haemoglobin concentrations in male athletes in comparison with the females (Sandbakk et al. 2012).

Numerous studies have investigated VO_2peak_ in different disciplines. The relative VO_2peak_ in sub-elite male runners has been shown to be 58.0 ± 8.3 ml kg^−1^ min^−1^, declining from 20 to 81 years (Kusy and Zielinski 2014). The young athletes in our endurance group had slightly higher values (60.8 ± 9.5 ml kg^−1^ min^−1^), indicating that they are quite well trained in their endurance-related disciplines. The elite kayakers (17.9 ± 2.7 years; 61.4 ± 4.4 ml kg^−1^ min^−1^) and canoeists (22.4 ± 5.5 years; 61.8 ± 4.0 ml kg^−1^ min^−1^) in the study by Buglione et al. (2011) possessed similar VO_2peak_ values compared with our athletes.

The VO_2peak_ in the sprint- and power-related athletes in the age group of 20–90 years (Kusy and Zielinski 2014) ranged from 30 to 60 ml kg^−1^ min^−1^ (mean ± SD: 47.0 ± 8.2 ml kg^−1^ min^−1^), with the highest values in the youngest athletes, which are similar to the values in our athletes (52.1 ± 7.7 ml kg^−1^ min^−1^ in males; 46.1 ± 7.2 ml kg^−1^ min^−1^ in females). In combat disciplines, aerobic capacity has been reported to be one of the most important physical factors for achieving good results. The VO_2peak_ of the junior combat athletes in this study are comparable with those from earlier investigations (male VO_2peak_: 55.6 ± 9.5 ml kg^−1^ min^−1^; female: 46.4 ± 5.2 ml kg^−1^ min^−1^). Wrestlers competing at national and international level have been shown to reach VO_2peak_ values of approximately 53–56 ml kg^−1^ min^−1^ (Yoon 2002). Several investigators found VO_2peak_ values in junior judo athletes as well as boxers of approximately 53–66 ml kg^−1^ min^−1^ (Franchini et al. 2011; Arseneau et al. 2011).

The VO_2peak_ values of our 12- to 13-year-old male and female tennis players were similar to the range documented by Fargeas-Gluck and Léger (2011) (54.2 ± 5.9 ml min^−1^ kg^−1^; recreational; 12.9 ± 0.3 years). When comparing with older racquet players higher and lower values of VO_2peak_, respectively, have been reported. Male adolescent badminton players (age: 21.3 ± 1.7 years) have been reported to have VO_2peak_ values that range from 50 to 70 ml kg^−1^ min^−1^ (Faude et al. 2007; Ferrauti et al. 2001; Kovacs 2007). Altogether, the VO_2peak_ values of our young racquet players (male: 54.9 ± 6.7 ml kg^−1^ min^−1^; female: 44.0 ± 4.8 ml kg^−1^ min^−1^) are in the same range as reported by previous studies involving elite adult racquet players.

In team sports such as basketball, field hockey and soccer, the assessment of physical fitness, including the VO_2peak_ measurement gained scientific interest because success in matches may be explained by the higher physical fitness levels of the players (Wisloff et al. 1998). VO_2peak_ values of three different Greek soccer divisions (~26 years) as well as Belgian soccer players ranged from 56 to 59 ml kg^−1^ min^−1^ (Ziogas et al. 2011; Boone et al. 2012). Younger soccer players (age: 13.5 ± 0.4 years) achieve VO_2peak_ values in a range of 55–59 ml kg^−1^ min^−1^ (Sperlich et al. 2011a). Lower VO_2peak_ values have been reported for volleyball (46.9–51.1 ml kg^−1^ min^−1^; 17.5 ± 0.5 years) (Duncan et al. 2006) and basketball (51.6 ± 5.7 ml kg^−1^ min^−1^, age: 15.8 ± 0.8 years and 50.3 ± 4.0 ml kg^−1^ min^−1^, 16.7 ± 1.2 years) players (Ignjatovic et al. 2011; Castagna et al. 2009).

In endurance-related events, athletes need to be able to perform for a long period of time at a high fraction of VO_2peak_ (Costill et al. 1973). In the past, several “thresholds” have been used to determine the link to the fractional utilisation of VO_2peak_ (Faude et al. 2009). In our opinion, the assessment of blood lactate at its first significant elevation above resting levels during the step test protocol (v_LT_) (Kindermann et al. 1979) and its corresponding % of VO_2peak_ are important key variables for determining the submaximal performance level of an athlete (Costill et al. 1973). During the incremental test protocol, the lactate-based threshold typically occurs between 50 and 70 % of VO_2peak_, while it is common for endurance athletes to have values of 80–85 % of VO_2peak_ (Sjodin and Svedenhag 1985). Based on our data, the v_LT_ and % of VO_2peak_ remain stable with increasing age in boys but decrease with age in girls. This occurrence can be attributed to the following two points: (1) children’s reduced glycolytic capacity (Eriksson 1972) and (2) their increased oxidative capacity (Eriksson 1972; Haralambie 1982).

In the past, the RE has been assessed to discriminate amongst athletes with respect to their endurance levels (Costill et al. 1973). Data regarding RE in different age, gender, and disciplines have not been collected extensively in the past. In elite distance runners, the RE has been shown to range between 45 and 60 ml kg^−1^ min^−1^ at a given speed (Davies and Thompson 1979). The RE ranged from 23 to 49 ml kg^−1^ min^−1^ in our male endurance athletes and from 21 to 62 ml kg^−1^ min^−1^ in the female athletes. Furthermore, running economy exhibited only slight differences between the different disciplines and remained constant across age.

Finally, a comparison of the v_peak_ values between studies is impossible due to the numerous different protocols used for the evaluation of cardiopulmonary tolerance testing.

## Limitations of the study

Although we were able to classify different important variables related to endurance in youth elite athletes some limitations are noteworthy. First, some of the measured parameters (i.e. maximal running speed and VO_2peak_) are protocol-dependent. In the present study the duration of each increment was 5 min to assure steady state conditions for oxygen uptake, blood lactate and heart rate during submaximal stages. This protocol has been applied in several other studies (Sperlich et al. 2010, 2011b; Abe et al. 2015) and a recent study confirmed that heart rate, end-exercise blood levels of lactate and RER do not differ between different incremental protocols, and maximum oxygen uptake is approx. 4.2 % lower compared to ramp testing (Sperlich et al. 2015). Based on the latter study we believe the present protocol reflects (1) a good compromise to determine several important endurance variables (at least VO_2peak_, v_LT_, % of VO_2peak_); (2) reduces the stress of frequent testing and (3) can be applied by coaches and athletes to quantify the (youth) athletes’ level of endurance performance (superior, excellent, poor, fair etc.). However we must accept that the data provided here are based on treadmill running and the values derived from incremental cycling or other type of exercise testing may vary.

## Conclusions

The current data classifies selected cardiopulmonary values of top-level athletes of different age, gender, and disciplines during a graded exercise running treadmill protocol. We could show that v_LT_ and % of VO_2peak_ remain stable over age in boys but decrease over age in girls in young elite athletes. Further, the running economy exhibited only slight differences between different disciplines and remained constant with age in youth elite athletes. The present classification may aid to assess cardiopulmonary tolerance during this type of exercise.
